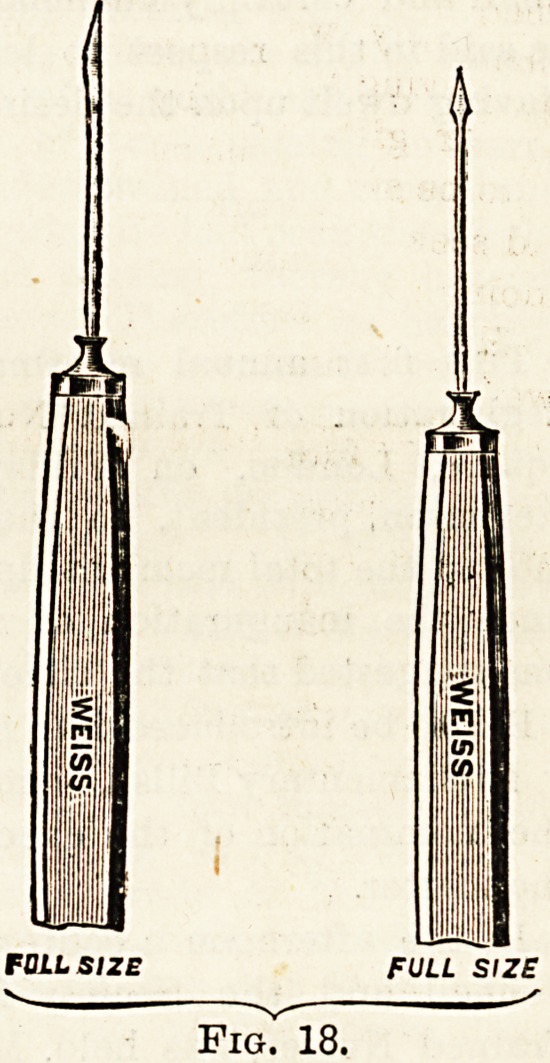# The Hospital. Nursing Section

**Published:** 1903-05-16

**Authors:** 


### The Hospital.

### Nursing Section.

Contributions for this Section of "The Hospital" should be addressed to the Editor, "The Hospital" Nursing Section, 28 & 29 Southampton Street, Strand, London, W.C.

No. 868.—Vol. XXXIV. *SATURDAY, MAY* 16, 1903.

### Notes on News from the Nursing World.

### IN MEMORY OF QUEEN VICTORIA.

On Saturday the Princess Christian opened the Suffolk Victoria Nursing Institute at Ipswich. Full particulars of the building have already been given in our columns. It is intended to be a memorial of Queen Victoria, and in one of the principal rooms there is a fine medallion of the late Queen carved in white marble by Countess Gleichen. The inscription below the latter is as follows: "In memory of our beloved Queen Victoria, and in promotion of the object so dear to her heart, of providing skilful and tender nursing for the sick and suffering, this house was acquired and adopted for the use of the Suffolk Nurses' Association, A.D. 1902—'I am among you as he that serveth.'" Princess Christian formally unveiled the medallion in the nurses' sitting-room. Upwards of 80 purses were presented to her and Princess Victoria of Schleswig-Holstein on behalf of the institute, and among the company present was Mr. John Martineau, the donor of the building. The Princesses inspected the Home under the guidance of Miss Hunt, the matron, who had previously presented her Royal Highness with a handsome bouquet.

### PRINCESS CHRISTIAN'S TRAINED NURSES.

The committee of Princess Christian's Trained Nurses have issued their sixteenth annual report to the subscribers. The staff at the end of 1902 consisted of the lady superintendent, the assistant superintendent, four Queen's nurses, including one midwife, twenty-one private nurses working from the home at Clarence Villas, and four Queen's nurses in the branch districts of Eton, Chertsey, Addlestone, and Egham. Two more houses were taken during the year, one being for the accommodation of the district nurses and to provide two sick rooms for the staff. Princess Christian most kindly furnished the nurses' sitting-room in this house. She also presented during the year special badges to eight nurses on completion of three years' service, and two nurses received gifts from the committee, one on the occasion of her marriage after four years' service, and the other on leaving for home duties after seven years. In the parishes of Windsor and Clewer 505 cases were nursed and 13,097 visits paid; in Eton the figures were 373 and 4,975; in Egham 63 and 2,073; in Addlestone 132 and 2,801; in Chertsey 72 and 2,259. Here, as elsewhere, the work of the midwife is steadily increasing, and a pupil nurse was taken for three months.

### NURSING IN THE NAVY.

With reference to the reports which have been current concerning a scheme for an increase of the nursing staff in the Navy, we understand that an official announcement will shortly be made. In the meantime it may be stated that the volunteers for the Auxiliary Royal Naval Sick Berth Reserve, which is to be established, will be taken chiefly from the St. John's Ambulance Brigade and the St. Andrew's Ambulance Corps, with as many trained male nurses as it is possible to obtain. Trained sisters cannot, of course, take duty on ships engaged in war, and at present the Reserve is for men only. But when further plans have been settled, it will probably include female nurses with full hospital training.

### A MUCH-NEEDED HOME FOR OPEN-AIR CASES.

On another page we publish a letter from Dr. Deanesley, recommending a house, "Bronwylfa," Penmaenmawr, North Wales, for private paying patients who need open-air treatment. We are informed that this house, which is under the management of Mrs. Mary White, late matron of the Wolverhampton Hospital, fulfils the conditions laid down by Dr. Kelynack in his paper on open-air treatment in a recent issue of The Hospital. It is far removed from any other building, is surrounded on three sides by fields and mountains, and the sea is immediately in front. There is ample accommodation in the grounds for pleasant open-air life, and the house is airy and sunny. The climate, too, of Penmaenmawr is found to be well suited to the open-air treatment, and nervous and anæmic patients especially do remarkably well there. There is so much difficulty and delay in procuring accommodation of this kind that we have much pleasure in calling attention to this establishment, having known Mrs. White for many years and having confidence that anyone who is fortunate enough to gain admission would be most comfortable and satisfied. Her terms are from two guineas to four guineas per week according to the requirements of each case; an extra fee is charged for a special nurse. Mrs. White has had special experience in the management of one of the best of English sanatoriums. All communications should be addressed to Mrs. White, Bronwylfa, Penmaenmawr, N. Wales. The letter from Dr. Deanesley will be found on page 125 of The Hospital.

### THE STATE REGISTRATION PANACEA.

According to one of the resolutions which was passed at a conference in London on Friday, the legal registration of trained nurses by Act of Parliament is "a matter of urgent national importance." If so, however, more than 736 out of the thousands of trained nurses in the United Kingdom would, surely, have joined the society for State registration. We do not think that the House of Commons will be inclined to seriously consider any measure which may be presented to them unless, or until, it has a much stronger backing than this. Moreover, with the conspicuous exception of Miss Isla Stewart, no matron of any great London hospital appeared at the meeting in support of the legislation which is said to be a matter of urgency. Miss Stewart's speech, holding out prospects of an "enormous increase in expenditure in the nursing staff" as the certain result of registration, is not altogether calculated to secure the end in view.

### HUDDERSFIELD GUARDIANS AND THE DEPARTMENTAL REPORT.

A special committee of the Huddersfield Guardians, with praiseworthy industry, presented a report of their own on the report of the departmental committee of the Local Government Board on nursing the sick in workhouses. This has since been issued in pamphlet form, and it is noteworthy for the suggestion that the Local Government Board should themselves set out a curriculum of training, hold examinations, and issue certificates. As to the recommendations of the departmental committee, they approve the proposal that a probationer trained in a minor school, after twelve months' training, should be called a qualified nurse; but dissent from the suggestion that nurses should be allowed "leave of three weeks in a year, one day a month, alternate Sundays, half a day a week, and two hours daily." They propose as an alternative that the question of leave should be left to the discretion of each individual board. They also advise that similar freedom should be given to each Board of Guardians to carry out, or set at nought, the arrangements recommended by the departmental committee with regard to the duties of the superintendent nurse. We regret that we cannot congratulate the Huddersfield Guardians upon the result of the labours of their committee. While they have blessed the scheme for bringing into existence an order of nurses who would be called qualified before they were properly trained, they have disparaged recommendations which we believe are calculated to improve the standard of nursing in Poor-law infirmaries.

### ROYAL SOUTH HANTS HOSPITAL.

The matron and nurses of the Royal South Hants and Southampton Hospital are watching with interest the erection of the handsome new home which they hope to enter at an early date. There are three sitting-rooms for the probationers on the ground floor—one with a bow window towards the street for the first and second year probationers, another smaller one facing the tennis-court for the third year probationers, and a study, which will be fitted with necessary books, for those who wish to read up quietly for their examination. On each landing there are box-rooms and linen-rooms, with an ample supply of bath-rooms. Each probationer will have a small bedroom for herself; and on the first floor there will be a sick-room for nurses off duty ill. The rooms of the night nurses are on the top floor, and a luggage lift will run from the top to the bottom of the building. It is satisfactory to learn that the Nurses' League in connection with the hospital, whose constitution and bye-laws are to come up for final discussion at the meeting in June, is thus early able to pay its way and support its journal.

### REORGANISATION OF CROYDON INFIRMARY STAFF.

The new order issued by the Local Government Board is involving very considerable changes in the nursing staff of Croydon Infirmary. At a meeting of the Board of Guardians on Tuesday, it was reported that the matron left on April 30th. The Board decided to meet on Tuesday next with the object of appointing her successor, who will be superintendent of nurses as well as matron, and fill other offices.

### THE MATRON OF JOHANNESBURG GENERAL HOSPITAL.

Mrs. Florence Magill, matron of Bradford Infirmary, has been appointed matron of the General Hospital, Johannesburg, at a salary of £300 a year. Mrs. Magill was trained at Crumpsall Infirmary. She then went to Liverpool, and has been connected with Bradford for the last 14 years. She is a woman of exceptional tact and charm, and will be a great loss to Bradford Infirmary, where she has inaugurated so much good work. The best wishes of many old friends will follow her to South Africa.

### PRIVATE NURSES AT THE ROYAL DEVON HOSPITAL.

The Governors of the Royal Devon and Exeter Hospital quote their report for 1902 as showing that the branch of private nurses is one of the best sources of the income of the institution. At the beginning of the year there was a balance of £98 due to the treasurer on the nursing account, and at the end of it the balance in hand was £55, notwithstanding that no less than £91 was paid out of the receipts for furniture at the Chaplaincy, which has been altered in order to afford accommodation for the private staff. It may be added that the hospital has always the first claim upon the services of the private nurses if they are needed in the wards.

### PROBATIONERS AT CAMBERWELL INFIRMARY.

Eighteen probationers who have been trained at the Camberwell Workhouse Infirmary received last week their certificates in the board room. Dr. Partridge, Chairman of the Infirmary Committee of the Board of Guardians, stated that the whole of them had passed, in a very creditable manner, an independent examination conducted by one of the physicians of Guy's Hospital. This is the first occasion on which such an event has taken place at Camberwell Infirmary.

### SALARIES AT WOLVERHAMPTON.

At the last meeting of the Wolverhampton Board of Guardians the question of the salaries of nurses was discussed. The house committee had previously gone into the matter, and it has been proposed that the salaries of the charge nurses be from £30 to £40, according to ability. An amendment that the salary be £30, rising £2 10s. to £35, was, however, carried, and the Board were invited to confirm the report of the committee. The Rev. G. W. Johnson urged that the maximum salary should be £40, but in the end a compromise was adopted; and henceforth the salary is to be as follows:—A charge nurse with less than two years' experience is to receive £30; with two years' experience, £32 10s.; and with three years' experience and upwards, £35.

### BOOKS FOR INFIRMARY NURSES.

A statement of some importance has been made by the Local Government Board upon the question whether it is legal for Poor-law guardians to contribute towards the purchase of books for the infirmary nurses. The view of the Board is that the legality of such expenditure would depend upon whether the books were reasonably required to enable the nurses to do their duty, and such as could not be expected to be provided by them. This, however, is a question which the Board leaves to the decision of the district auditor in the first instance, and they are not prepared to express any opinion prior to the matter being brought before him. We hope that the opportunity suggested will be speedily given. There are books which it would be most useful for the infirmary nurses to have at their command for study and reference, and it would be a great advantage to obtain the sanction of the Local Government Board to a grant on the part of the guardians sufficient to provide them.

### NORWOOD COTTAGE HOSPITAL.

Last year at the annual meeting of the Norwood Cottage Hospital Dr. Galton raised a discussion on the alleged long hours of probationers in certain London hospitals, and suggested that Mr. Tritton, M.P., the President of the hospital, should mention the subject in Parliament. At the annual meeting on Saturday Dr. Galton referred to what then took place, and said that the discussion was reported in The Hospital and commented upon as "more unfounded charges." It was not, he said, expedient for him to follow these charges up at the time, but he was glad that he did call attention to them, as he knew that improvements had since taken place in the hours of work, food, and on other points. Mr. Tritton, M.P., said that they had to deplore the loss of their matron, Miss Henderson. She had endeared herself very greatly to everyone connected with the Norwood Cottage Hospital, and her resignation was very much regretted. They wished her every happiness in her married life. They had been fortunate, he thought, in securing their present matron, Miss Clark. The committee had every confidence that under her able management and supervision the Norwood Hospital would continue to prosper. Subsequently, Dr. J. Sidney Turner moved that the thanks of the meeting be given to the matron and the nursing staff for their continued kindness and attention to patients. Dr. H. Hexley seconded and the resolution was very cordially passed.

### ASYLUM WORKERS' ASSOCIATION.

The annual report of the Asylum Workers' Association, to be presented at the meeting this week, shows that for the twelve months ending December 31, 1902, there were 119 life members, 119 associates, and 4,664 ordinary members, making a total of 4,902, as compared with 4,116 on the roll of 1901. During 1902 members were elected from Ashwood House, Boreatton Park, East Sussex County Asylum, Fife and Kinross District Asylum, Herts County Asylum, and Normansfield, these institutions not being primarily connected with the Association. We have already mentioned the award of the gold and silver medals, and we notice that in the report the Executive Committee state that in consequence of the unexpectedly large number of entries of competitors at the lower periods of service they have been compelled to increase the minimum length of service for competitors for gold medals to 35 years instead of 30, and for silver medals 30 years instead of 25. The financial statement shows that the receipts, including a balance from last year, amounted to £470, and payments to £401.

### AN OPENING IN WEST AFRICA.

Several correspondents from various parts of the country write in reference to our remarks as to an opening in West Africa for a mission nurse, to express their desire to take advantage of it. Any further information can be obtained from Miss Mary Elms, C.M S. Medical Mission, Onitsha, Southern Nigeria, West Africa. We have no doubt that the Church Missionary Society, Church Missionary House, Salisbury Square, E.C., would answer inquiries upon the subject.

### RECREATIONS FOR NURSES.

The second number of the *Chelsea Infirmary Nurses' Journal* contains the *precis* of a paper read by the president at the first meeting of the debating society in connection with the League, on "The Best Forms of Recreation for Nurses." Some excellent advice is given in respect to the annual holiday, when there is time for a spell of recreation, and the president suggested that one of the most pleasant plans, for those who can afford it, is to travel abroad and see new lands. But she warns nurses wherever they go not to be too energetic or tear and rush about, trying to crowd as much as possible into each day. As to the recreation of off-duty time, she wisely recommends that as much of it as possible should be spent in the open air; and points out that even the drive on the top of a 'bus can be most reviving and destructive of cobwebs.

### LONDON SPECTACLE MISSION SOCIETY.

The issue of the tenth annual report of the London Spectacle Mission Society is a reminder of the excellent work done by the unobtrusive organisation which was founded by the late Dr. Waring in order to provide aged and poor persons whose sight is failing with glasses for their relief. How much the operations of the society are appreciated may be gathered from the fact that the number of applicants for help has grown from 531 in 1893 to 1,920 in 1902. In 10 years there have been upwards of 10,000 applications. Subscribers of five shillings receive four spectacle cards entitling four persons to relief. The address of the hon. secretary, Miss Waring, is 197 Sutherland Avenue, W

### SHORT ITEMS.

At an inquest held last week on the body of Emma Bowerman, described as a hospital nurse, and attached to a home in Devonshire Street, London, a verdict of suicide during temporary insanity was returned. The deceased had taken a quantity of carbolic acid and died on the way to the Middlesex Hospital.—Miss Esther Young's series of articles headed "A Few Words of Advice to Nurses" lately published in our columns, have been issued in the form of a shilling pamphlet, and may be obtained from the Scientific Press, 28 and 29 Southampton Street, Strand, London, W.C.

### The Nursing Outlook.

" From magnanimity, all fear above;
From nobler recompense, above applause,
Which owes to man's short outlook all its charm."

### THE LIVING WAGE OF THE DISTRICT NURSE.

FROM time to time absurd advertisements appear demanding the most perfect district nurse for the most inadequate wage; when these advertisements bring few or useless replies the usual rule is to lower the requirements rather than to raise the rate of pay.

Now the nurse is worthy of her hire, and the time has arrived when a strong stand should be made against the attempt to secure district nurses for what is not a living wage. We have had a revolution in the pay, the work and the feeding of the hospital nurse; we have seen co-operation more than double the earnings of the private nurse, and now we want to rescue the district nurse from the clutches of the pseudo-philanthropists whose niggard charity blesses neither those that give nor those that take. For most assuredly the under-paid nurse is the bad nurse, and her ministrations are of little benefit to her patients. In asking, therefore, the organisers of district nursing to give their nurses a living wage, we are asking them also to do their best for the poor cottagers; we are pleading not only for the nurse but for the patient.

When the vicar's wife is worried by the parish doctor to start a district nurse, she quite naturally asks first and foremost what the nurse will cost. The raising of money for a charity, the going round and begging from the rich is very unpleasant work, and a great deal of such duty falls on the clergy and their families. The great desire to get everything cheap, to be very economical, is strong in those on whom are many calls; and £10 less for the district nurse may mean £10 for the clothing club. And so the nurse is often advertised for at the very lowest sum, and some damp or dreary rooms are thrown in as mitigation of the offence; £30 and lodgings is not an infrequent offer, or £50 inclusive. Now a nurse cannot live at a proper standard of health and cleanliness, with a proper return on her time given to training, and with a proper provision for her old age, on £1 a week.

The district nurse needs to be very highly skilled, for great responsibilities may fall upon her in the doctor's absence, or, if the doctor decides on some operation, in his presence. In the country an operation case cannot be at once removed to a hospital as in a town, and the difficulty of making arrangements in a cottage and of carrying on the nursing treatment are very great. Also obstinate cases of rheumatism, and long cases of cancer can only be kept comfortable and cleanly in their village homes by good judgment and great skill. Of the maternity cases, of the emergencies that the nurse or midwife may have to meet there, it is not so necessary to speak. And for the introduction of sanitary and hygienic reforms, which go to the prevention of disease, not only is there knowledge needed, but also the tact and politeness that mark the real gentlewoman. Practically a district nurse needs to be of good education and social status, pleasant in her manner and patient in her character; she should have had three years' training as a nurse, including six months' special midwifery and six months' district nursing. At the outset of her career she should not be over 30 years of age, and during the whole of her working days she needs active vigour and unexceptional health. That is what is wanted; and the standard must be maintained as well as obtained.

This standard cannot be maintained on less than £70 a year. For even in a village where rents are low, two bright rooms and a little bit of garden are not to be had for nothing, and the nurse must live in cleanly, hygienic rooms (for example is more powerful than precept), and must have such bright surroundings as will help her to maintain her physical and mental health. Again, though vegetables and certain bodily foods may be cheap in the country, books and lectures and papers are specially dear and difficult, and knowledge must not be allowed to rust. Moreover, the expensive uniform must be kept as trim and as tidy as in London; there must be no stinting of clean aprons because the boots and cloaks suffer from the rough outdoor work. A nurse's laundry bills should always be large. Then, at least £10 a year should be put into the National Pension Fund, and money is needed for holidays, and possibly for a bicycle.

We would seriously ask all organisations, especially village associations, which are the chief offenders, to think of these things, and to remember when they have their nurse not to neglect her or let her get dull. Society and sympathy are specially necessary to the lonely worker, and the snobbishness that refuses them to a woman because she is a worker, is luckily becoming a thing of the past. Yet only last week a nurse committed suicide, and gave as one of her reasons that she "had no one to talk to." An appeal to employers is only one side of the question: there is also an appeal to nurses. If nurses would refuse cheap and nasty training, if they would refuse cheap and nasty posts and aim for district work at being Queen's nurses, all would be well. As a matter of fact most district nurses nowadays do belong to the Queen Victoria Jubilee Nursing Institute, have fulfilled its conditions, and are, in return, receiving good wage and protection, and the social position that comes from belonging to a recognised society. If all district nurses could be drawn into this fold we should be sure that all were receiving a living wage. But we know only too well that there are a number of worthy women toiling in lonely parishes on mere pittances, and that these women cannot make provision for their future, cannot maintain the proper standard of living, must be growing rusty in their knowledge and more incapable year by year. It seems easy at first to slip through a brief training, it seems possible at first to live on a poor salary, but as years go on the hopelessness and the wickednesses of the situation disclose themselves and condemnation falls on employer and employed alike. Even if nursing be charity, still its practice is a profession, and it ought to be well paid and well performed.

### Lectures on Ophthalmic Nursing.

By A. S. Cobbledick, M.D., B.S.Lond., Senior Clinical Assistant Royal Eye Hospital, late House-Surgeon and Registrar, Royal Eye Hospital.

LECTURE X.—INSTRUMENTS IN COMMON USE (*cont.*).

(1) *Knives.* These are very numerous. The chief are:—(*a*) *Cataract* knives. Graefe's (Fig. 5). This is the knife in most common use for making a linear incision, whether for cataract extraction or for an iridectomy for glaucoma. Other cataract knives are Beer's (Fig. 6). (*b*) *Iridectomy* knives (Figs. 7 and 8). These are sometimes called "bent broad needles." *Bowman's* (Fig. 9). (*c*) Tarsal cyst knife and scoop (Fig. 10).

(2) *Scissors.* (*a*) Strabismus. These are blunt-pointed and have short blades. (*b*) Iridectomy scissors may have

Fig. 5.

Fig. 6.

Fig. 7.

Fig. 8.

Fig. 9.

Fig. 10.

Fig 11.

Fig. 12.

Fig. 13.

Fig. 14.

the blades set at an angle or curved on the flat. De Weeker's (Fig. 14), are very useful, but require more care than ordinary scissors.

(3) *Forceps.* Fixation (Fig. 15), cilia (Fig. 16), iris (Fig. 17).

(4) *Needles.* Cataract (Fig. 18).

FULL SIZE FULL SIZE

FIG. 15.

FULL SIZE

FIG. 16.

FULL SIZE

FIG. 17.

FULL SIZE FULL SIZE

FIG. 18.

Other instruments will be mentioned in the following sections.

Most ophthalmic instruments require careful handling, on account of their slender make and the all-important necessity of their points being in good order. They must be kept in aseptic glass cases, fitted with special racks, so arranged as to prevent the points coming in contact with any part of the case.

All instruments must be sterilised by immersion in boiling water before use; ordinary tap-water, with soda added, may be used, but it is much better to use distilled water, *i.e.*, water free from all salts; there is no doubt that the use of distilled water prevents instruments from losing their fine point and edge. An immersion of two or three minutes is quite sufficient; the instruments should then be placed in a tray containing sterilised water or a solution of carbolic acid 1 in 40. It is not absolutely necessary for instruments to be placed in lotion after they have been boiled; a useful and simple method is to place them on an aseptic handkerchief—rendered so by boiling in water or subjecting it to steam sterilisation—spread over a clean plate or dish; the end of the handkerchief may then be turned over the instruments so as to protect them until the operator requires them. After operation they should be washed in cold tap-water, all traces of blood removed, and again placed in the steriliser for five minutes; they should then be carefully dried and returned to the instrument case.

### East End Mothers' Home.

THE annual meeting of the East End Mothers' Home was held at the home on Friday last week, Dr. Owen Lankester in the chair. In the course of a brief speech he said that during the past year they had found that the accommodation for nurses was insufficient, and had in consequence taken premises opposite for the purpose of supplying this deficiency. This departure would, at the very lowest estimate, cost them from £600 to £700, as in addition to furnishing and fitting up the new premises they hoped to turn the quarters vacated by the nurses into wards, which would involve structural alterations. The Chairman appealed to those present not only to give help themselves towards this object, but to endeavour to interest their friends in the work of the home.

The Countess of Jersey moved the adoption of the report, and said that her knowledge of the East End was chiefly confined to the children. But she had plenty of experience of the poor in country districts, and knew well what a debt of gratitude they owed to such institutions as this, which trained those who rendered such valuable service as district nurses. It was in London itself where such homes as these were most needed. In the future they might hope that with the advance of science contagious diseases would be abolished, hereditary complaints greatly checked, and even the fatal results of accidents averted, but for all time institutions such as the East End Mothers' Home would be required. The poor mothers in their hour of need had great

### EAST END MOTHERS' HOME—*Continued.*

claims upon humanity, and those who had been confided to the care of such a home as this went forth into the world again happier and better than before; for it was not only medical skill and good nursing that they found, but also love and kindness. They learnt there lessons of health and cleanliness which enabled them to bring up their children under improved and healthier conditions.

The Hon. G. J. Goschen, M.P., in seconding the motion, said that it was always difficult to make a financial appeal, and more especially at this time, when appeals were so incessant and met with such willing responses; but there was one view of the question which they might press successfully in asking for subscriptions. There were many people who though not wealthy were charitably disposed, but often refrained from giving to the greater charities either because they feared their gifts were not worth offering or because they seemed to be swamped by the larger donations. Such people should seek out some small and deserving institution which was doing good work and to which every shilling was of benefit. He maintained that the East End Mothers' Home answered the description of such a charity: it was small and certainly financially struggling; in fact it might be said in this respect to lead a hand-to-mouth existence. Having dwelt upon the desire felt by all connected with the home to give a quiet and happy time to the poor women who came under their care, he concluded by bearing testimony to the excellent work done by the staff and to the desire which animated them all to give the best work they could to the home.

Dr. William Gow, in moving the re-election of the Board of Management, said he had lately inspected the home and was struck by the very valuable work that was being done. The board were labouring under the disadvantage of occupying modified dwelling-houses, but the total results were extremely good. He pointed out the difficulties under which the poor in great towns lived, and said it was a most remarkable fact how few hospitals of this kind existed in London. All the four hospitals devoted to this purpose were founded in the middle of the eighteenth century, and when in 1870 it was proposed to add to their number the scheme met with a great deal of opposition. He was of opinion that this opposition was very wise, because until comparatively lately these hospitals had not been safe for the patients on account of the little regard paid to sanitation.

The Rev. E. R. Ford seconded.

The report gave the total of in- and out-patients for 1902 as the highest on record, and the financial statement showed a small margin on the side of receipts.

### The State Registration of Nurses.

THE first annual meeting of the Society for the State Registration of Trained Nurses was held at 20 Hanover Square, London, on Friday morning last, Miss Louisa Stevenson, president, in the chair. The report presented showed the total membership to be 736, 275 having joined since the inauguration of the Society in May, 1902. It was suggested that the time had arrived for the drafting of a Bill to be introduced into the House of Commons, and that a Parliamentary Bills Committee should be appointed, upon the nomination of the executive committee, to deal with the matter.

In the afternoon a conference, convened by the Matrons' Council and the Society for the State Registration of Trained Nurses, was held, Miss Stevenson again presiding. In her opening remarks she pointed out that it was of supreme importance to the public that nurses should be competent and trustworthy. At present, unfortunately, the private nursing world was more largely exploited by untrained and unsuitable persons than any other branch of nursing work, and the public paid the fees commanded by trained and experienced nurses for the services of women who were neither the one nor the other. When it was realised that between the visits of the medical attendant the nurse was left in sole charge, and that the comfort and even the safety of the patient depended upon her care and devotion, it would be seen that the stake which the public had in the question could not be exaggerated. The only safeguard, in her opinion, was State registration, which she believed would benefit every section of the community, and not least the nursing profession itself. Of the 80,000 "nurses" going about the country only a comparatively small number were entitled to be called "trained," while the faith of an all-confiding public in a uniform and a pair of white sleeves was astonishing. This arose from the fact that there was no minimum standard of training: every hospital was a law unto itself. The idea that every woman by virtue of her womanhood was capable of ministering to the sick had by no means died out, and this sentimental notion accounted for a state of things otherwise inconceivable.

Miss Isla Stewart, matron of St. Bartholomew's Hospital, dealt with the probable results of legislation on general hospitals. The term "trained nurse" would be defined: it would connote a definite period of study, a definite time spent in the wards, and the passing of an examination set by qualified examiners outside the hospital. She thought that the hours would also be shortened. These measures would imply an enormous increase in expenditure on the nursing staff. The hospital authorities would then have to determine how much of the money subscribed for the benefit of the sick could be spent on the training of the nurses. This would probably bring about a reduction of the payment to probationers, with possibly the institution of a premium, since their value would tend to decrease. There would, however, be a corresponding rise in the value of the fully trained nurse. Such subjects as could not be practised in the wards, *e.g.* cooking, massage, and maternity nursing, would, she thought, be acquired in a preparatory school, and all subjects that did not directly benefit the patients would have to be paid for by the nurse. There would be an increase in theoretical instruction, and she foresaw a danger lest practical work should suffer. This must be kept in view by the framers of the proposed Bill.

Miss Eleanor C. Barton, matron of Chelsea Infirmary, claimed that the training given in many Poor-law infirmaries entitled them to recognition by the central authority when constituted; and Mrs. Matthews, late matron of the Grove Fever Hospital, Tooting, advocated a system of affiliation between general and special hospitals with a view to complete the training of nurses under the proposed conditions.

Mrs. Bedford Fenwick urged that the proposed board should consist of members elected by the nurses themselves: it should be a self-governing body. She regretted that England was behind the Colonies in the matter of legislation for nurses, and stated that Acts providing for registration had just been passed in New York and Illinois.

Miss Janet Speed, a registered nurse from New Zealand, spoke of the advantages of State protection, and outlined the provisions of the Act which came into force in 1902.

Miss Rogers, matron of the Leicester Infirmary, Miss Poole, matron of the East Lancashire Infirmary, Bolton, Miss Mollett, matron of the Royal South Hants and Southampton Hospital, having spoken, resolutions were then unanimously passed in favour of legislation providing for the registration of nurses.

### Dr. Downes on Workhouse Nursing.

THE thirty-fourth annual conference of Poor Law Guardians for the West Midland district was held at Malvern on Tuesday and Wednesday.

Dr. Downes, of the Local Government Board, read a paper on "Nursing in Workhouses, and the Report of the Departmental Committee," in the course of which he submitted for discussion some main points of the report of the Departmental Committee appointed last year by the President of the Local Government Board. The questions before the committee were:—

1. As to any difficulties in obtaining an adequate supply of nurses.
2. As to the qualifications of nurses.
3. As to the respective spheres of duty of masters, matrons, and superintendent nurses.

The business of the committee was to ascertain facts and to suggest remedies.

Within five years there had been an increase of something like 44 per cent. in the number of paid officers engaged in nursing the indoor sick poor in England and Wales, and this advance had continued; for at the end of 1902 the total number of such officers had risen to 5,566—representing an increase of no less than 52 per cent. on the numbers of 1896.

Not the least satisfactory feature of this increase in the number of paid nurses was the fact that it had been spread generally over the kingdom, and had indeed been greatest in those workhouses which were classed in the return as "Country Workhouses."

The instructive returns annually compiled by the inspectors for their respective districts told the same tale of continuous increase in the number of nurses, and decline in the number of pauper ward attendants.

So much then had actually been accomplished notwithstanding the difficulties, and he attached great importance to this first point established by the inquiry, regarding it as substantial evidence of the determination of guardians generally throughout the country to provide for their sick and aged poor. But satisfactory as this was, it by no means proved the absence of difficulty, and even serious difficulty, in obtaining nurses. The verbal evidence obtained by the committee was supplemented by inquiry addressed to every union in England and Wales, and the conclusion of the committee was that the difficulties, though substantial, were by no means so widespread as had been asserted, inasmuch as fully three-quarters of the workhouses and infirmaries proved to be admittedly free from difficulty even at a time when the demands of the war and of epidemics had exceptionally limited the supply of nurses. The difficulties, he said, were not such as to justify wholesale interference with a movement of steady advance, and guardians possessed sufficient powers for dealing with many of them. The provision of comfortable quarters, varied food, sufficient recreation for the nurses and not overworking them, would help guardians to obtain and retain suitable nurses, and no one who realised the strain of sick nursing would question the reasonableness of such concessions to the comfort of the nurses.

The committee was of opinion that the permanent staff should be adequate to cope with night nursing, and that extra nurses should be obtained from an institution when necessary. Dr. Downes next reviewed the committee's recommendations as to the grades and qualifications of nursing officials. One recommendation was that there should be at least one trained nurse, preferably qualified in midwifery, resident in every workhouse. The grades of nurse employed by guardians would remain practically the same, but the qualifications for each office were defined, the standard was raised, and the conditions of employment were specified. The central point of the whole was the recommendation that definite qualifications should in future replace the indefinite requirements of Article II. of the Order of 1897. He had lived long enough to learn that reforms of this kind, if they were to be real and lasting, must be effected gradually, and in a system of local government must progress *pari passu* with the march of public knowledge and sentiment. The requirement of 1897, indefinite and unsatisfactory as it might be, was as far as the Local Government Board could go at that stage; but the remarkable advance indicated by the statistics already given proved the soundness of that Order, and fully justified the present proposals of the committee.

Dr. Downes referred to the great increase in the number of probationers.

It was to the probationer movement that the committee looked for the main supply of Poor Law nurses, and perhaps one of the most important parts of the report was the scheme of training schools. These they divided into major and minor. As to the former, there was nothing essentially new, but the committee recommended a defined curriculum where there was at present none under the Order, and stipulated for a final examination by two independent examiners, of whom one should be a lady, herself the head of a recognised training school. Some considered that a standard number of beds should replace the requirement of a medical officer (or assistant medical officer) giving his whole time to the guardians' service. Something might be said for a minimum standard of beds, but he would deprecate the omission of the medical officer. He had found by experience that the most economically managed infirmaries were those which had the whole-time services of a medical officer. The second grade of training school in the committee's scheme was the "minor training schools." He believed that in no way could they more effectually dispel stagnation and quicken the life of a workhouse sick ward than by bringing into it a class of nurse coming to learn the first steps in their calling under trained and competent women whose heart was in the work. He had seen the whole administration, both medical and nursing, thereby braced and stirred into renewed efficiency. Therefore he heartily supported the proposal to organise the so-called "minor" schools. He said so-called because, if he compared them with a great number of the cottage and general hospitals of this country which receive probationers, he regarded them as anything but "minor." If the voluntary hospitals could produce a sufficient and suitable supply of nurses for all Poor Law requirements, and if the nursing profession were graded and organised, as some day he hoped it might be, they need not now be troubling themselves as to conditions of training or differences of nursing rank; and the regulating authority in Poor Law matters need simply require that the qualification for this or that office should correspond with a definite grade of the nursing world. But that time had not arrived, technical education had not yet grasped its obligations, nor was it probable that the voluntary hospitals, whose proper work daily passed more and more to the Poor Law, would ever meet the nursing needs either of the workhouses or the public at large.

And so it came that the workhouses must train their own, and there was a demand that the Local Government Board should for the time fill the place of an educational authority.

There was a desire on the part of some leaders of the nursing world to obtain a recognised status for their calling, but he felt that they would overshoot the mark if they insisted on the exclusion of the humbler rank of nurse. Neither could the great mass of the community afford to pay highly trained nurses; were they therefore to have no nursing aid?

He was convinced that it was quite possible for the minor schools to produce a class of nurse most useful either for the workhouse or the cottage, and well-grounded in the essential rudiments of her profession; and he was confident that in this the guardians might serve not only the interest of their own sick but the good of many besides. If such schools were established, they would be an encouragement to local women to acquire nursing knowledge.

Dealing with that portion of the committee's recommendations in regard to the duties of superintendent nurses and their relations to the master and matron, Dr. Downes said no detailed rules had been laid down. It should be sufficient to say that the duty of a nurse was to nurse and to obey the lawful directions and regulations of the guardians. The keynote of the committee's recommendations was to trust the guardians, and to strengthen their hands by consolidating and adding to the good work already begun.

### Everybody's Opinion.

[Correspondence on all subjects is invited, but we cannot in any way be responsible for the opinions expressed by our correspondents. No communication can be entertained if the name and address of the correspondent are not given as a guarantee of good faith, but not necessarily for publication. All correspondents should write on one side of the paper only.]

### WORDS OF ADVICE TO NURSES.

"AN EVERY-DAY SORT OF NURSE" writes: As Miss Young's advice to the nursing profession seems to have started a correspondence, I think it is perhaps worth while to say that, while fully appreciating the wisdom of Miss Young's remarks with reference to a nurse's "duty," especially in private work, I thought at the time that in a nurse's off-duty time she ceases to be professional—at least she ought to cease—and that her way of dressing, etc., ought not to be subject to her professional but to her social standing. After all, nurses are not "nuns," and surely we are none the less nurses that we take our ordinary share of outing and pleasant recreation with our own friends. Miss Young's kindly and practical words of advice will, I think, be a help to many of us in our work.

### GIFTS TO NURSES.

"HARD-WORKING" writes: I should like to thank an "English Nurse" for her excellent letter on "Gifts to Nurses," as I agree with every word she writes, and was also much upset at the scathing remarks aimed at the private nurse a few weeks ago in the *Nursing Section.* Some of the happiest moments of my life have been when choosing a souvenir for some dear one. Even from a small child, when one buys an utterly useless article, the memory of the giving remains in one's mind for years. What would have been our feelings if it had been returned? Rich and poor patients have their feelings of appreciation and gratitude, if not of affection, and I cannot imagine how nurses can possibly refuse a gift if it is offered to them in a kindly spirit. Looking back ten years ago I wonder whether it would have been more difficult to decline to accept the china teapot from a poor mother, given to me from the little second-hand furniture shop (where I was nursing), with the wish that I should use it always and think of them; or the lovely silver one presented to me a few weeks ago by a Countess. Somehow we private nurses always seem to have unkind remarks aimed at us, when I am sure we need help and encouragement more than any of our sisters, for private nursing is at the best trying, and it needs a purely unselfish life for its duty.

### Presentations.

BIRKDALE HOSPITAL.—Miss Laura Matthews who was appointed nurse-matron by the Urban District Council of Birkdale about two years ago, has resigned on account of her approaching marriage. At a meeting of the Birkdale Health Committee held last week, it was decided, to present Nurse Matthews with a cheque for special services rendered during the time she has had charge of the Birkdale Hospital.

READING UNION INFIRMARY.—Miss P. Pendrey who is leaving Reading Union Infirmary to take up an appointment as district nurse, has been presented by the sister and nursing staff with a handsome dressing case, and by the master and matron with a gold-mounted umbrella.

ROYAL HALIFAX INFIRMARY.—Miss Eveleen Dane has been presented by the matron, sisters, and nurses of Royal Halifax Infirmary with a silver Queen Anne tea service, and by the maids with a silver and pearl paper knife, on the occasion of leaving to take up duties as matron of Drumcondra Hospital, Dublin.

### Appointments.

[No charge is made for announcements under this head, and we are always glad to receive, and publish, appointments. But it is essential that in all cases the school of training should be given.]

BARTON-UPON-IRWELL UNION.—Miss A. M. Kenyon has been appointed charge nurse. She was trained at Rochdale Union Infirmary.

CARMARTHENSHIRE INFIRMARY.—Miss Clara Moore has been appointed staff nurse. She was trained at Burton-on-Trent Infirmary and Nottingham Isolation Hospital.

CHALMERS HOSPITAL, EDINBURGH.—Miss Nora Smythe has been appointed sister. She was trained at the Great Northern Central Hospital, London, and has been sister at Croydon General Hospital. She holds the L.O.S. certificate.

CHELTENHAM GENERAL HOSPITAL. — Miss Beatrice Stoneham has been appointed sister. She was trained at Wolverhampton General Hospital, and has since been sister at the Suffolk Hospital, Bury St. Edmunds, and sister at the District Hospital, Grimsby.

CHIPPENHAM INFIRMARY.—Miss Maud Schafer has been appointed head nurse. She was trained at St. George's Infirmary, Fulham Road, London, and has since been nurse at Newton Abbot Infirmary. She holds the L.O.S. certificate.

CRAIG CONVALESCENT HOME FOR CHILDREN, MORECAMBE.—Miss Valetta Shout has been appointed matron. She was trained at the Alexandra Hospital for Children with Hip Diseases, Queen's Square, London, and Addenbrooke's Hospital, Cambridge. She has since been sister at the City Hospital, Liverpool, and the Hull Sanatorium, and matron at the Sanatorium, Morecambe.

EXETER INFECTIOUS DISEASES HOSPITAL.—Miss Marian Jones has been appointed matron. She was trained at the Bath Royal United Hospital, and has since been charge nurse and night superintendent in the City of London Hospital, Victoria Park, and senior sister at the Borough Hospital, Croydon.

HENDON SICK ASYLUM.—Miss Catherine Fitzmaurice has been appointed charge nurse. She was trained at the City of London Infirmary, Bow Road, E., where she was afterwards charge nurse. She has also done private nursing, and holds the L.O.S. certificate.

NORTH DEVON INFIRMARY, BARNSTAPLE.—Miss M. Jones has been appointed charge nurse. She was trained at Ashton-under-Lyne Infirmary, and has done private nursing in Nottingham.

PLUMSTEAD INFIRMARY.—Miss Leah Gabriel has been appointed ward sister. She was trained at Toxteth Infirmary, and has since been night charge nurse at Brook Hospital, London, and sister at St. Giles's Infirmary, Camberwell.

POPLAR AND STEPNEY SICK ASYLUM.—Miss Nellie Alexander has been appointed superintendent night nurse. She was trained at the Western Infirmary, Glasgow, and has since been nurse at the South-Eastern Hospital, New Cross, and Tonbridge Union Infirmary.

SWANSEA DISTRICT NURSING ASSOCIATION.—Miss Lilian Trendell has been appointed superintendent nurse. She was trained at the West London Hospital, Hammersmith, and York County Hospital, and has since been Queen's Nurse at Gosport.

TIVERTON INFIRMARY AND DISPENSARY.—Miss Agnes McElney has been appointed matron. She was trained at Edinburgh Royal Infirmary.

WARGRAVE DISTRICT NURSING ASSOCIATION.—Miss P. Pendrey has been appointed nurse. She was trained at the Reading Union Infirmary, and afterwards held the post of charge nurse and head charge nurse for five years at the same institution. She holds the L.O.S. certificate.

### Echoes from the Outside World.

### The Royal Visit to Scotland.

THE King and Queen left London on Monday morning for Scotland. They drove in a closed carriage to King's Cross owing to the threatening weather. An Newcastle an address was presented, to which the King replied to the Mayor and Corporation that he regretted the halt in their ancient and renowned city was necessarily so short. The royal party arrived at Dalkeith House shortly before seven, being welcomed by the Duke and Duchess of Buccleuch. They had been met at the station by Lord Balfour of Burleigh and the Lord Provost of Edinburgh. The latter offered to the King the keys of the city, and the King returned them, stating that they could not be in more loyal hands. Enormous crowds had assembled in the streets, and remained late into the evening to witness the giant bonfire, which, unfortunately, burnt itself out too speedily to please the expectant people. On Tuesday the citizens were early astir so as to line the nine miles which separate Dalkeith from Holyrood, where the levée and drawing room were to be held. It is 80 years since a Court was held at the Scottish palace, and 300 years since a king and queen visited Edinburgh together. Their Majesties drove in semi-State, and soon after their arrival the levée commenced. This was followed by a Court. The rooms in which these functions were held have some lovely samples of Gobelins and Dutch tapestry, and in one chamber a suite of needleworked furniture, said to have been the work of Mary Stuart.

### A Royal Betrothal.

ON Monday it was officially announced that an engagement had been entered into between her Serene Highness the Princess Alice, eldest daughter of his Serene Highness the Prince and her Grand Ducal Highness the Princess Louis of Battenberg, and his Royal Highness the Prince Andrew, younger son of their Majesties the King and Queen of the Hellenes. Princess Alice, who was born on February 25th, 1885, is the elder daughter of her parents, and is a grandniece of the King and Queen of England. Prince Andrew, who was twenty-one last February, is a nephew of King Edward and Queen Alexandra. His Royal Highness arrived in London from the Continent on Friday evening, and visited the King and Queen at Buckingham Palace on Sunday. In the evening both he and his *fiancée* dined with the Prince and Princess of Wales at Marlborough House, their Majesties being also of the party.

### The Duke of Cambridge.

NOTWITHSTANDING his eighty-five years, the Duke of Cambridge takes his due share in performing public functions. On Wednesday last week he opened the Earl's Court Exhibition, and in proposing success to the exhibition the Duke said that its influence would be felt all over the country, with general advantage to the community. On Sunday morning he attended the annual thanksgiving service in the Chapel of the Foundling Hospital. The children of the hospital sang the music of the "Te Deum" and the "Benedictus" beautifully, and in the prayer the name of the benevolent old shipmaster, Thomas Coram, who founded the school 164 years ago, was reverently remembered. The Bishop of Kensington preached the sermon. After the service the Duke walked to the schoolroom, where he presented rewards and testimonials for good conduct to former inmates of the hospital, boys and girls, who have recently come of age. His Royal Highness expressed the pleasure it gave him to come amongst them once again to hand rewards to the recipients, and he fervently thanked God that he could still perform that office. He hoped that the young people whom he saw before him, who had been well and carefully educated, would lead honourable and useful lives, which would not only be satisfactory to themselves, but would redound to the credit of the Foundling Hospital.

### Fire Exhibition at Earl's Court.

THE Exhibition at Earl's Court this year is of a particularly attractive character. With the exception of an Arab village—which is a well-arranged novelty—the interest centres in matters connected with fire. The collection of old and new fire appliances should on no account be missed. It includes the London squirts and buckets of the sixteenth century, the old hand fire-engines of the seventeenth and eighteenth centuries—including one entitled the "Deluge," which was actually used at the Great Fire of London 1666—and, lastly, the well-equipped machines of the present day, amongst which are placed a motor steam fire-engine and a petrol motor-driven chemical engine and combined fire-escape. There is also a show of old engines and modern inventions from the United States, Holland, France, and other countries, a side-show with fine scenic effect of the Great Fire of London, with St. Paul's in flames, and another pourtraying the eruption of Mont Pelée and its disastrous results. The performance, which is given twice daily in the Empress Theatre, will probably delight many visitors. In the first part is a procession of firemen of all epochs dating back to the days of the Roman soldiery, and a drill tournament between fire brigades; but the second portion of the programme "Fighting the Flames," is the more sensational. Here is a busy thoroughfare with pedestrians and passing vehicles; then a cry of "Fire" is raised, screams are heard, and flames are seen coming from the burning building; the alarm is given, the police hold the crowd in check, the engines arrive, living persons are rescued—and at last the conflagration is subdued. The whole is wonderfully realistic.

### The Royal Academy.

THOUGH there is no work in the Royal Academy this year which can be called "the picture of the year," there is much that is of interest. Mr. Orchardson's "Mrs. Siddons in the Studio of Sir Joshua Reynolds," commands attention at once. The great actress, clad in white, is reciting before a distinguished company, which include Sir Joshua himself, Burke, Macklin, Mrs. Jordan, Sheridan, etc. Most of the portraits are very good, but the artist has not altogether followed the recognised likenesses of Mrs. Siddons. The picture is small, but instinct with life. Another canvas of special interest is the Westminster Abbey Coronation Ceremony of Mr. Bacon. The artist has chosen the moment when the Archbishop is doing homage to his Sovereign, and the King is leaning anxiously forward to help the half-fainting prelate. The incident is treated with much artistic discretion. Mr. Charles Furse has an imposing group of a horseman "Returning from a Ride," a pretty woman walking by his side. Never has this clever artist been better represented. Other portraits worthy of notice are "Mrs. Huth Jackson," by F. D. Millet; "The Rev. Nevison Loraine and his Lurcher Sirdar," by Mr. Briton Rivière—no loving dog was ever more faithfully pourtrayed—and "The Lady Evelyn Cavendish," by Mr. John S. Sargent. Professor Herkomer also has a vivacious portrait of Gen. Baden-Powell. Sir Edward Poynter contributes some storm nymphs, and Sir Alma-Tadema, a picture of some fish in a marble tank, watched by three of his Greek damsels. The Chantrey Trust have made four purchases, namely, Mr. David Murray's "In the Country of Constable," and Mr. Adrian Stokes' Alpine landscape "Autumn in the Mountains;" with two pieces of sculpture "Remorse," a poetic figure, by Mr. H. H. Armstead, and a spirited group of children, "The Springtide of Life," by Mr. W. Robert Colton, one of the newest A.R.A.'s. Last year no purchases were made by the Chantrey bequest.

### For Reading to the Sick.

UPWARD AND ONWARD.

Higher! higher to aspire!
That is all my soul's desire,
Nearer to the Light and Love
In which saints and angels move,
Nearer to the Glorious Throne,
And to Him Who sits thereon,
To perfection nigher, nigher
To my Saviour, higher! higher!

Higher! higher! every thought
More into His presence brought!
Every passion, every feeling,
More His inner Life revealing,
Less of self from hour to hour,
More of faith's transforming power,
Yearnings Heaven-ward that aspire
Unto Jesus, higher! higher!

Higher! Higher! till at length
Passing on "from strength to strength,"
Pressing up from grace to grace,
I behold that long'd-for Face,
Which is daily o'er me leaning,
With its deep and tender meaning,
And doth into light retire,
But to lead me higher, higher!

*Monsell.*

In the possibilities of the ever-increasing advancement of our renewed life in other worlds, lies one of our truest encouragements, when we mourn the slowness and imperfections of our progress in this world. Our greatness is not so much in what we here attain, but in what we may attain hereafter. And so our trust for the present is not so much the actual gain, but our tendency towards a future gain. The possible reach of grace is too great to be compassed by any present rule. The measure of the stature of Christ is too vast to suppose that any present attainment can be adequate to the conception realised. All is now "in part." "Now we know in part and we prophecy in part." Only, "when that which is perfect is come, then that which is in part shall be done away."

We judge of the future by the tendencies of the present. The upward growth will be according to the bent of the strengthening stem. We cannot see God, but we can see what tends towards God. The mystical ladder's ascending steps are within our gaze. The Form of the Everlasting, Who standeth above, is shrouded in the inscrutable darkness. We are at best like tropical plants struggling beneath ungenial skies with stunted growth, which can bear no fruit, nor expand into flower, but which, if transplanted into the regions of the sun, would develop into richest foliage and abundant produce.

The poor deformed races of men who creep along the frozen seas, if removed to the sunny south might rise to a nobler stature and developed powers. This same law nourishes Christian hope, through the belief that the faint feeble beginnings of this season of struggles and fears, while the corruptible body weigheth down the soul, far off from God, when transferred to more genial skies shall, if not here, yet there, expand into their predestined fullness, that all whose eyes shall then behold God, shall grow into the perfect likeness of God, in the power of the vision of God.—*T. T. Carter.*

### Notes and Queries.

**REGULATIONS.**

**The Editor is always willing to answer in this column, without any fee, all reasonable questions, as soon as possible.**

**But the following rules must be carefully observed :—**

1. **Every communication must be accompanied by e name and address of the writer.**
2. **The question must always bear upon nursing, directly or indirectly.**

**If an answer is required by letter a fee of half a-crown must be enclosed with the note containing the inquiry. We cannot undertake to forward letters addressed to correspondents making inquiries unless a stamped envelope is enclosed.**

*Passage to Canada.*

(63) 1. Can you tell me how to get my passage paid to Canada in return for services ? 2. Is there a Nursing Institute in British Columbia ? 3. Can you tell me a Canadian paper to advertise in ?—*Ethel A.*

1. Only by advertising in the daily papers. 2. Perhaps the matron of the Provincial Royal Jubilee Hospital, Victoria, B.C., might be able to help you. 3. Write to Sell's Advertising Agency, Fleet Street, E.C.

*Queen Alexandra's Imperial Nursing Service.*

(64) Will you kindly tell me to whom I must apply for particulars concerning Queen Alexandra's Imperial Nursing Service ?—*A. R. A.*

Write to the Matron-in-Chief, 68 Victoria Street, S.W.

*Hospital Training.*

(65) I am anxious to have a three years' general training in a London hospital where salary is paid from the first. Will you kindly advise me where to apply ? I have had no previous experience.—*B. V. D.*

You cannot do better than consult "The Nursing Profession: How and Where to Train." Full particulars are given as to the terms and conditions of hospital training both in London and in the provinces.

*Midwifery. Massage.*

(66) Will you kindly tell me (1) Where a course of midwifery can be taken in or near Southampton, but not in London ? (2) Where can lessons for electrical treatment be taken, and what time is required for the course ?—*Nurse Bessie.*

1. There is no public school for training midwives in Southampton, but doubtless if you were to write to the Secretary of the Midwives' Institute, 12 Buckingham Street, Strand, W.C., she would give you advice. 2. Do you mean a course of massage with applied electricity ? Make inquiries of the Dowsing Institution, 28 York Place, Baker Street, W.

*Nursing Institute in the South.*

(67) Will you kindly give me the address of the Hollond Institute, and the names of any other nursing associations in the South of Europe or at Algiers ?—*Lina and Nurse Margaret.*

The Hollond Institute is now the Nice Nursing Association, and the address is Villa Pilatte, Avenue Désambrois. Other nursing institutions in the south of Europe are the English Nurses' Institute and Medical Home, Sunny Bank, Via Borgo Pescio, San Remo. The Association of Trained Nurses and Masseuses, 7 Via Rondinelli, Florence, the Anglo-American Nursing Home, 265 Via Nomentana, Rome, and the British Cottage Hospital, Alger Mustapha, Algiers.

*Children's Nurses.*

(68) I should be glad of the address of the institution in London where ladies are trained as children's nurses ?—*D. R.*

The Norland Institute, 10 Pembridge Square, London, W.

*Maternity.*

(69) The fees mentioned under this heading, in answer to *Eunice* last week, were for the training of midwives, not of monthly nurses. The latttr generally are about half the former.

**Important Nursing Textbooks.**

"The Nursing Profession: How and where to Train." 2s. net; 2s. 4d. post free.

"A Handbook for Nurses." (New Edition). 5s. net; 5s. 4d. post free.

"The Human Body." 5s. post free.

"Ophthalmic Nursing." (New Edition). 3s. 6d. net; 3s. 10d. post free.

"Gynæcological Nursing." 1s. post free.

"Art of Feeding the Invalid." (Popular Edition). 1s. 6d. post free.

"Practical Hints on District Nursing." 1s. post free.

## Figures and Tables

**Fig. 5. Fig. 6. Fig. 7. Fig. 8. Fig. 9. Fig. 10. Fig 11. Fig. 12. Fig. 13. Fig. 14. f1:**
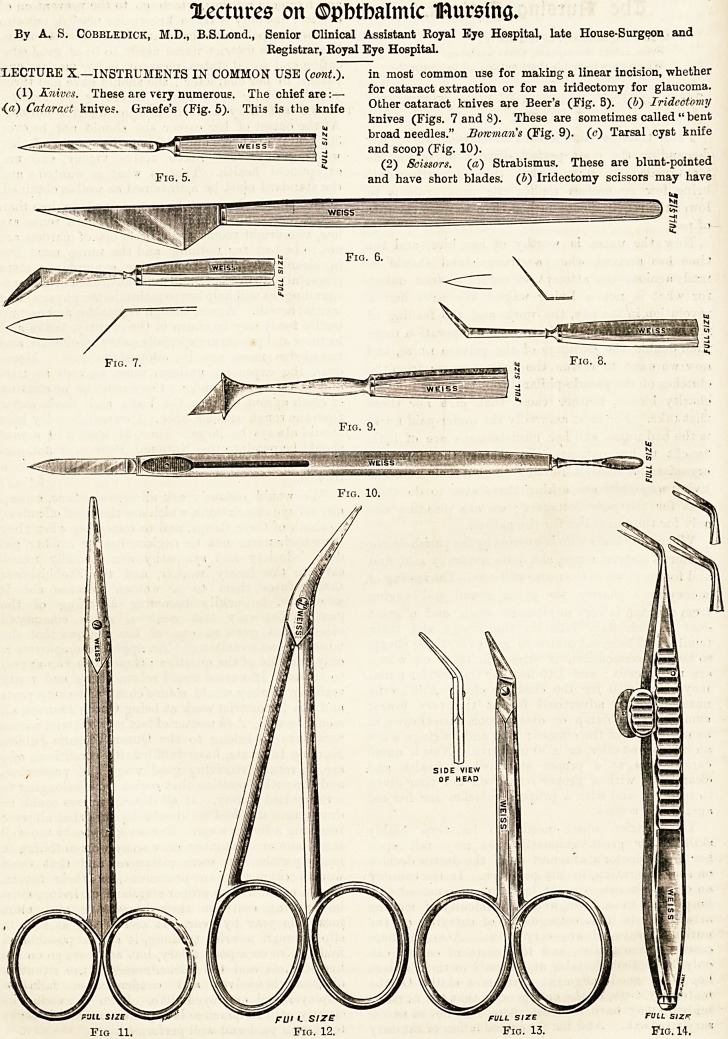


**Fig. 15. f2:**
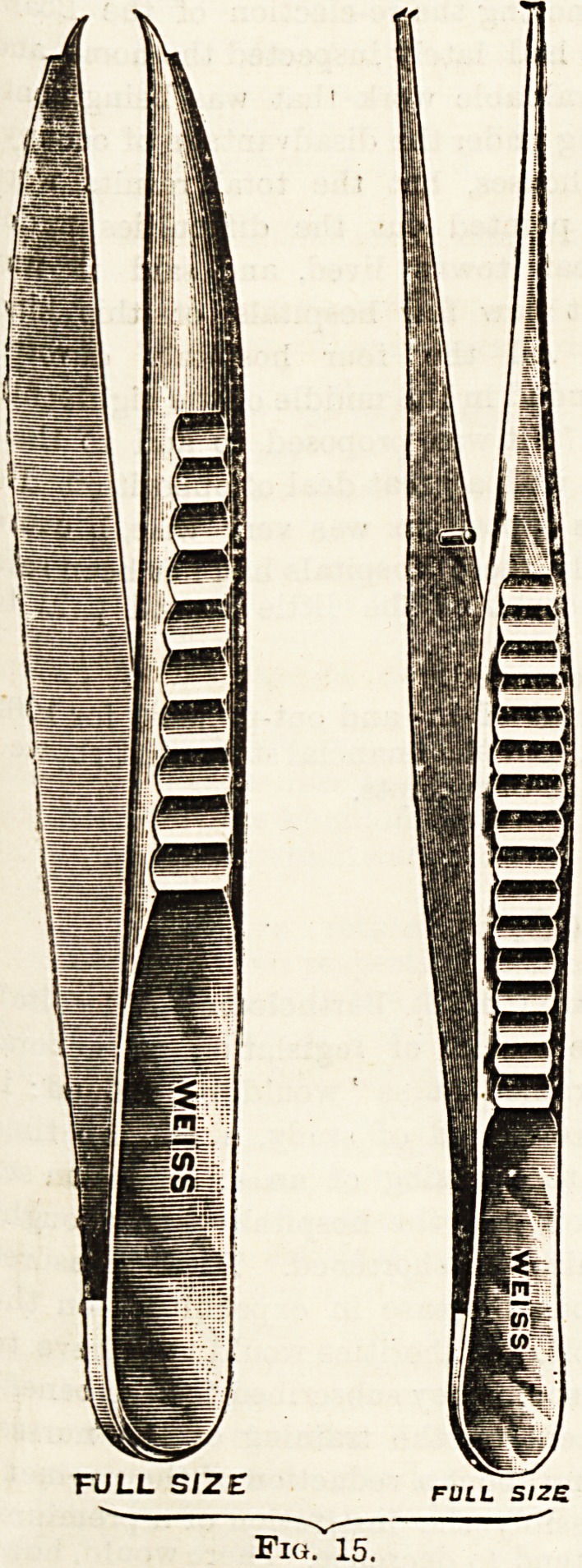


**Fig. 16. f3:**
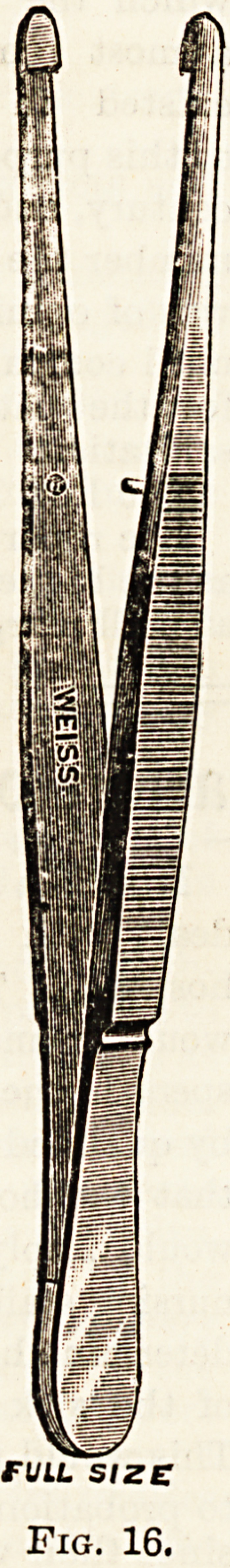


**Fig. 17. f4:**
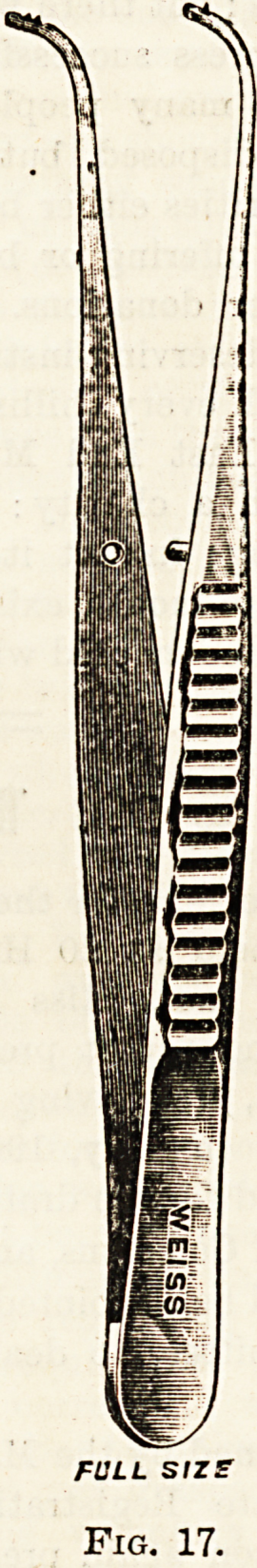


**Fig. 18. f5:**